# QiShenYiQi Pill Improves Myocardial Hypertrophy Caused by Pressure Overload in Rats

**DOI:** 10.1155/2021/5536723

**Published:** 2021-06-16

**Authors:** Shichao Lv, Qiang Wang, Meifang Wu, Meng Li, Xiaojing Wang, Ling Xu, Junping Zhang

**Affiliations:** ^1^The First Teaching Hospital of Tianjin University of Traditional Chinese Medicine, Tianjin 300193, China; ^2^Tianjin Key Laboratory of Traditional Research of TCM Prescription and Syndrome, Tianjin, China

## Abstract

Pressure-overloaded myocardial hypertrophy is an independent risk factor for various cardiovascular diseases (CVDs), such as heart failure (HF), arrhythmia, and even sudden death. It is reported that QiShenYiQi pill (QSYQ) is widely used in the treatment of CVDs and can prevent pathological hypertrophy of myocardium, but its specific mechanism is still unclear. In this study, a rat model of myocardial hypertrophy was established through the pressure overload caused by abdominal aortic constriction in Wistar rats. The rats were randomly divided into model group, valsartan group, and QSYQ group, and sham-operated animals served as the control group. At the 4 and 8 weeks of intervention, the general morphology of the heart, myocardial collagen content, collagen volume factor (CVF), collagen type I, collagen type III, myocardial pathological changes, and the expression of ANP, *β*-MHC, TGF-*β*1, and CTGF were analyzed, respectively, in order to explore the possible effect of QSYQ on the mechanism of myocardial hypertrophy. We observed that QSYQ could effectively improve myocardial hypertrophy in pressure-overloaded rats, which was related to the regulatory mechanism of TGF-*β*1 and CTGF.

## 1. Introduction

Myocardial hypertrophy is cardiomyocyte hypertrophy and myocardial fibrosis, which is a compensatory and adaptive response to pressure overload [1]. However, the myocardium is usually overloaded, so it has maladaptive reactions and can be used as an independent risk factor for various cardiovascular diseases (CVDs), such as heart failure (HF), arrhythmia, and even sudden death [2]. Pathological left ventricular hypertrophy (LVH) can be seen in hypertension, aortic stenosis, and other CVDs. Hypertension is the main cause of pressure overload myocardial hypertrophy with a prevalence of 20%–30% [3]. At the same time, hypertension combined with LVH can increase the incidence of acute myocardial infarction, congestive heart failure, sudden death, and other cardiovascular events by 6–8 times [4, 5]. The reconstruction of cardiac structure caused by hypertension is mainly LVH. LVH is an adaptive change of chronic cardiac pressure and volume overload and is a common complication of hypertension. The early compensatory performance is manifested as centripetal hypertrophy, and the late decompensation is manifested as centrifugal hypertrophy, and then the cardiac function decreases and eventually leads to HF [6]. At present, the mechanism of LVH has not been fully elucidated, and its pathogenesis involves hemodynamic factors, neurohumoral regulation factors, cardiovascular tissue autocrine and paracrine factors, and genetic factors [3]. All kinds of drugs may improve LVH in varying degrees through effective hypotension, but the mechanisms and effects of different drugs on LVH are different. Studies have shown that the five antihypertensive drugs have different effects on reversing LVH: angiotensin II receptor antagonists, calcium antagonists, angiotensin-converting enzyme inhibitor (ACEI), diuretics, and *β*-blockers can decrease the left ventricular mass index of patients by 13%, 11%, 10%, 8%, and 6%, respectively [7]. Modern medicine for LVH mainly focuses on renin angiotensin aldosterone system, among which ACEI, angiotonin receptor blocker, and aldosterone antagonists have been studied most, but only for a single pathological connection. Traditional Chinese medicine has a variety of ingredients, which can act on multiple targets at the same time to regulate different action links, and has the advantage of comprehensive regulation of LVH treatment.

QiShenYiQi pill (QSYQ) is made of effective components of *Radix Astragali, Radix Salviae Miltiorrhizae, Radix Notoginseng,* and *Lignum Dalbergia Odorifera*, which was approved by the China State Food and Drug Administration for the treatment of CVDs in 2003 [8, 9]. The effective ingredients of QSYQ are extracted by modern preparation technology, with stable dosage form and controllable quality [10]. Studies have shown that QSYQ can significantly improve the degree of myocardial fibrosis in rats with abdominal aorta coarctation [11], reduce myocardial hypertrophy in rats with coarctation of aorta [12, 13], relieve left ventricular remodeling in rats with ligation of left anterior descending coronary artery [14, 15], and improve cardiac function and myocardial structure in rats with ischemia-reperfusion [16]. In addition, QSYQ can also effectively improve myocardial damage in adriamycin-induced cardiomyopathy mice [17, 18] and improve cardiac remodeling in rats with autoimmune cardiomyopathy [19]. However, the effect of QSYQ on myocardial hypertrophy is still unclear. In this study, a rat model of cardiac hypertrophy was established through narrowing the abdominal aorta to further explore the effect and mechanism of QSYQ on myocardial hypertrophy.

## 2. Methods

### 2.1. Animals

Male Wistar rats, weighing 180–200 g, were provided by the Laboratory Animal Center of the Academy of Military Medical Sciences (Beijing, China; certificate number SCXK (Jun) 2012–0004). Wistar male rats were raised in cages with constant temperature of 22 ± 2°C, a 12-hour light-dark cycle, free drinking water, and standard diet. All research procedures were in compliance with the Guidelines for the Care and Use of Laboratory Animals (NIH Publication no. 86–23, revised 1996) and approved by the Animal Ethics Committee of Tianjin University of Traditional Chinese Medicine, China (no. TCM-LAEC2016016).

### 2.2. Animal Model of Pathological Myocardial Hypertrophy

Abdominal aorta constriction method was used to establish myocardia hypertrophy model in pressure-overloaded rats [20]. In short, rats were anesthetized with pentobarbital sodium (45 mg/kg, intraperitoneal injection) and then fixed on the operating table supine, and a longitudinal incision in the abdominal cavity was performed under aseptic conditions. The abdominal aorta was exposed, and the abdominal aorta above the branch of the right renal artery was separated. The no. 7 needle was placed parallel to the abdominal aorta and ligated with the no. 4 surgical sutures. The needle was removed and 200,000 U of penicillin was instilled to prevent infection, and the abdominal cavity was closed. At the same time, a small amount of picric acid solution was applied around the incision to prevent wound infection in rats. After the operation, the rats were fed with sugar–salt solution and continuously injected intramuscularly with 200,000 U/d penicillin for 3 days to prevent infection. Rats in the sham operation group were operated on, and the sutures were placed without ligation. The remaining steps were similar to those in the surgery group.

### 2.3. Groups and Dosing

Four weeks after operations, rats with sham operation and aortic stenosis were randomly divided into sham-operated control group (intragastric infusion with equivalent distilled water), model group (intragastric infusion with equivalent distilled water), the valsartan group (intragastric infusion at 7.2 mg/kg; Novartis Pharma Ltd., Beijing, China), and QSYQ group (intragastric infusion at 135 mg/kg; Tasly Pharmaceutical Group Co., Ltd., Tianjin, China). The intervention was performed at 2 time points (4-week and 8-week intervention), with 8 rats in each group at each time point. The rats in each group were dosed once every morning.

### 2.4. Measurement of Myocardial Collagen Content

The content of hydroxyproline in myocardial tissue was determined by alkaline hydrolysis, and hydroxyproline kit (#A030-2) was from Jiancheng Biology Engineering Institute, Nanjing, China. Myocardial collagen contained 13.4% hydroxyproline. The myocardial collagen content was equal to the hydroxyproline content multiplied by 7.46.

### 2.5. Morphological and Histological Analysis

The rats were anesthetized by intraperitoneal injection of 3% pentobarbital sodium (45 mg/kg) after drug intervention for 4 weeks and 8 weeks. The heart was fixed in 4% paraformaldehyde overnight, routinely dehydrated, transparent, embedded, and sectioned, 5 *μ*m thick; deparaffinized, dehydrated using an ethanol gradient, and subjected to hematoxylin-eosin (H&E) staining and Masson staining (all from Baihao Biological Technology Co., Ltd., Tianjin, China). Then, it was dehydrated with ethanol gradient, washed with xylene, fixed with neutral resin, and observed under an optical microscope. H&E staining was used to estimate cardiac hypertrophy, while Masson staining was used to measure the area of collagen. The Image-Pro Plus image analysis software was used to randomly select 5 fields of view from each slide to measure the area of collagen. The average was calculated as the collagen volume factor (CVF) for this myocardial tissue. CVF = area of myocardial collagen fiber/total area of the image.

### 2.6. Western Blot (WB) Detection

The protein lysate was added to the myocardial tissue placed on ice for 30 min and centrifuged to extract protein. The protein concentration was determined by BCA method. The protein was separated using sodium dodecyl sulphate-polyacrylamide gel electrophoresis (SDS-PAGE) and transferred to polyvinylidene fluoride (PVDF) membranes. PVDF membrane was incubated with 5% skimmed milk powder at room temperature for 2 hours. Primary antibodies including collagen I antibody (dilution 1 : 1000) (#ab255809, Abcam), collagen III antibody (dilution 1 : 500) (#ab6310, Abcam), and *β*-actin antibody (dilution 1 : 5000) (#20536-1-AP, Proteintech Group, Inc., USA) were added and incubated at 4°C overnight. Then, the membrane was incubated with secondary antibody (dilution 1 : 5000) labelled by horseradish peroxidase (HRP) (#SA00001-2, Proteintech Group, Inc., USA) at room temperature for 2 hours. After the ECL luminescent reagent was coloured, the automatic gel imaging system was performed by exposure imaging. Image lab software was used to detect the expression of protein bands, and the relative expression of target protein was calculated with *β*-actin as the internal reference.

### 2.7. Real-Time Quantitative Polymerase Chain Reaction

Ultrapure RNA extraction kit (*#CW*0581, ComWin Biotech Co., Ltd., Beijing, China) was used to extract total RNA from the myocardial tissue. The HiFi-MMLV cDNA first-strand synthesis kit (*#CW*0744, ComWin Biotech Co., Ltd., Beijing, China) was used for reverse transcription, and the UltraSYBR Mixture with Rox (*#CW*0956, ComWin Biotech Co., Ltd., Beijing, China) was used for amplification. The primers for the target genes and household genes were purchased from Guangzhou Fulengen Co., Ltd., Guangzhou, China, including TGF-beta 1 (*#RQP*050181), CTGF (*#RQP050397*), and GAPDH (*#RQP*049537). In addition, the forward ANP primer was 5′-TCAAGAACCTGCTAGACCAC-3′, and the reverse ANP primer was 5′-GACCTCATCTTCTACTGGC-3′. The forward *β*-MHC primer was 5′-CATCATCACCAGAATCCAG-3′, and the reverse *β*-MHC primer was 5′-CTCTGCGTTCCTACACTCC-3′. The forward *β*-actin primer was 5′-CTGAACGTGAAATTGTCCGAGA-3′, and the reverse *β*-actin primer was 5′-TTGCCAATGGTGATGACCTG-3′. The 2^−△△Ct^ method was used for relative quantitative analysis of the raw data of RT-qPCR determination.

### 2.8. Immunochemical Analysis

The expression of TGF-*β*1 protein and CTGF protein in rats was measured by immunochemical assay. The slides were routinely dewaxed by xylene and hydrated by gradient ethanol, and the antigens were repaired with microwave. Then, an appropriate amount of hydrogen peroxide was added to block the endogenous peroxidase activity of the protein. A primary antibody (rabbit anti-rat IgG) was added and incubated at 4°C overnight. Furthermore, the slides were incubated with biotinylated goat anti-rabbit IgG. Finally, DAB was used for color rendering and hematoxylin was used for secondary dyeing (antibody against TGF-beta 1 (#BA0290), antibody against CTGF (#BA0752-1), streptavidin–biotin complex with peroxidase (SABC-POD) (rabbit IgG) ready-to-use kits (#SA1022), and DAB Chromogenic Reagent Kit (#AR1022) were all from Boster Biological Engineering Co., Ltd., Wuhan, China). After dehydration, transparent sealing was carried out with neutral resin. Determination of results: cells with clearly defined structures, significantly greater coloration than those with background coloration, and brown-yellow particles in the corresponding area were considered to be positive. The cells that did not develop any color or had the same level of coloration as the background were considered to be negative. We randomly selected 5 views from each slide under a 40 × fold optional microscope. The area ratio of the positive staining substances was determined by using the Image-Pro Plus imaging analysis software, and the average was calculated.

### 2.9. Statistical Analysis

All experimental results were statistically analyzed using SPSS software (v11.5; SPSS Inc., Chicago, IL, USA) and expressed as mean ± SD. One-way analysis of variance was used to compare multiple groups, and then the least significant difference (LSD) test was used for multiple comparisons. The value of *P* < 0.05 was considered statistically significant.

## 3. Results

### 3.1. Effect of QSYQ on Rat Cardiac Morphology

**Figure 1:**
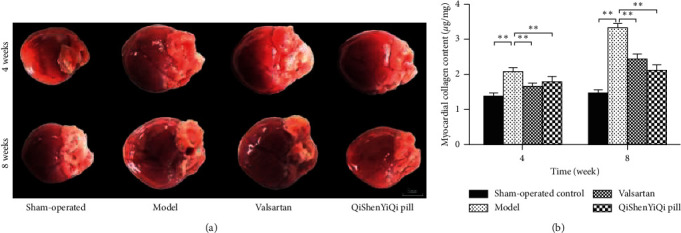
Effect of QSYQ on rat cardiac morphology. (a) The morphological changes in the heart of rats. (b) The myocardial collagen content of each group. Data are expressed as mean ± SD. ^*∗∗*^*P* < 0.01.

Compared with the sham-operated control group, the heart was significantly enlarged and myocardial collagen content increased significantly in the model group (*P* < 0.01) and increased with time. In contrast with the model group, the degree of cardiac enlargement and myocardial collagen content in the valsartan group and QSYQ group was reduced (*P* < 0.01), and it further reduced with the extension of the intervention time (Figure 1).

### 3.2. Effect of QSYQ on Myocardial Histopathology

**Figure 2:**
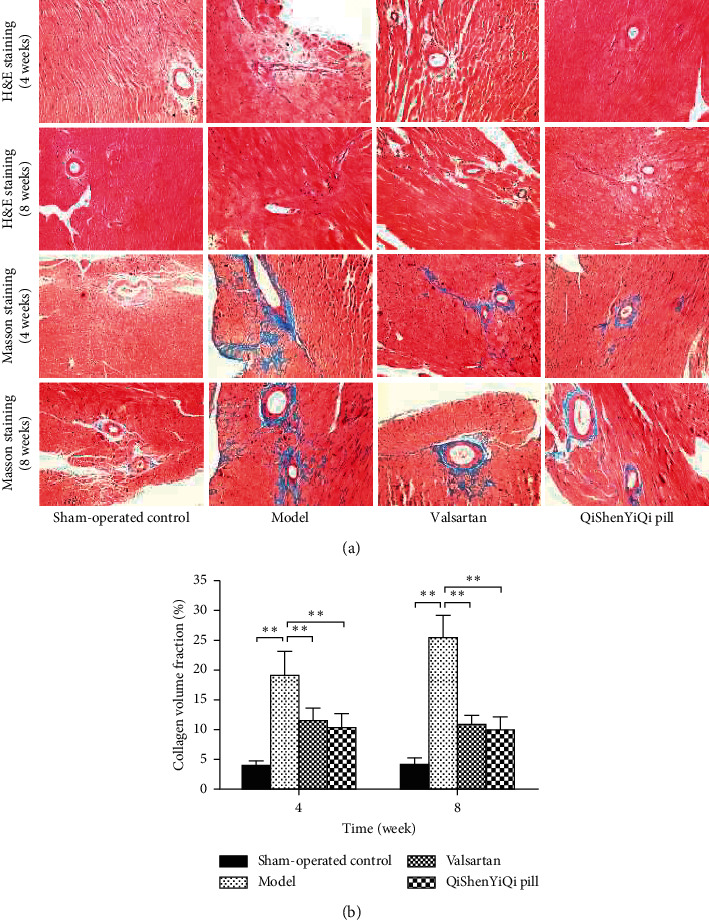
Effect of QSYQ on pathomorphism of myocardium. (a) Representative photomicrograph of hematoxylin and eosin (H&E) and Masson trichrome staining of myocardium. (b) The collagen volume fraction for each group. The collagen volume fraction was quantitatively analyzed by using Image-Pro Plus 6.0 (magnification, × 100). Data are expressed as mean ± SD. ^*∗∗*^*P* < 0.01.

H&E staining of myocardial tissue showed that myocardial cells were arranged orderly, and the cytoplasmic staining was uniform without necrotic foci in the sham-operated control group. But the model group showed extensive and multifocal myocardial fibrosis, hypertrophy and swelling of cardiac myocytes, proliferation of adjacent fibrous tissue, and inflammatory cell infiltration around the vessels. Compared with the model group, the above pathological changes were reduced in the valsartan group and the QSYQ group (Figure 2).

Masson trichrome staining of myocardial tissue showed that there were no obvious blue collagen fibers in the myocardial interstitium in the sham-operated control group, but in the model group, a large amount of collagen was deposited in the myocardial interstitium and around blood vessels, and the blue fiber area was larger. And the collagen volume fraction (CVF) of the model group increased significantly (*P* < 0.01), showing a trend of increase over time. After treatment (valsartan and QYSQ), the area of blue collagen fibers was smaller than that of the model group, and the CVF was significantly decreased (*P* < 0.01), indicating that the degree of myocardial fibrosis was reduced (Figure 2).

### 3.3. Effect of QSYQ on the mRNA Expression of ANP and *β*-MHC

**Figure 3:**
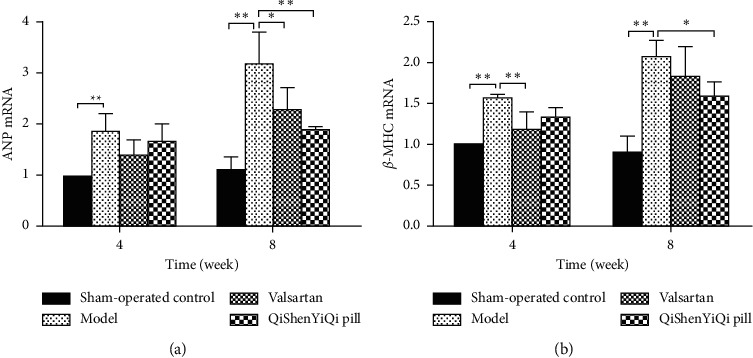
Effect of QSYQ on the mRNA expression of ANP and *β*-MHC. (a) The ANP mRNA expression for each group. (b) The *β*-MHC mRNA expression for each group. Data are expressed as mean ± SD. ^*∗*^*P* < 0.05 and ^*∗∗*^*P* < 0.01.

Compared with the sham-operated control group, the expression of ANP and *β*-MHC in the myocardium of rats mRNA was increased in the model group (*P* < 0.01) and further increased over time. As for the treatment groups, valsartan reduced the expression of *β* -MHC mRNA at 4 weeks and ANP mRNA at 8 weeks. However, QSYQ only reduced the expression of ANP and *β* -MHC mRNA at 8 weeks (Figure 3).

### 3.4. Effect of QSYQ on the Protein Expression of Collagen Type I and III

**Figure 4:**
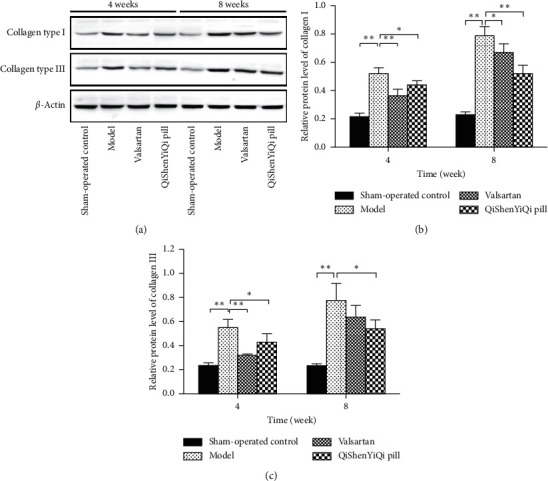
Effect of QSYQ on the protein expression of collagen type I and III. (a) Collagen type I and III expression in the myocardium of rats as detected by western blot. (b) The relative protein level of collagen type I in the myocardium. (c) The relative protein level of collagen type III in the myocardium. Data are expressed as mean ± SD. ^*∗*^*P* < 0.05 and ^*∗∗*^*P* < 0.01.

Compared with the sham-operated control group, the expression of type I and III collagen in the model group were upregulated (*P* < 0.01), and further increased over time, and increased again at 8 weeks. With regard to the two treatment groups, the expression of type I and III collagen was downregulated in both the valsartan and the QSYQ groups after 4 weeks. The valsartan only downregulated the protein expression of type I collagen, but QSYQ could downregulate both type I and III collagen at 8 weeks (Figure 4).

### 3.5. Effect of QSYQ on mRNA Expression of TGF-*β*1 and CTGF

**Figure 5:**
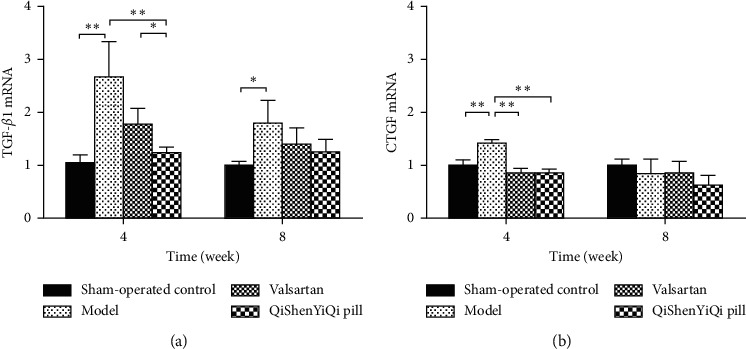
Effect of QSYQ on the mRNA expression of TGF-*β*1 and CTGF. (a) The TGF-*β*1 mRNA expression for each group. (b) The CTGF mRNA expression for each group. Data are expressed as mean ± SD. ^*∗*^*P* < 0.05 and ^*∗∗*^*P* < 0.01.

At 4 weeks after abdominal aortic coarctation, pressure overload caused an increase in the expression levels of TGF-*β*1 and CTGF mRNA in the rat myocardium (*P* < 0.01). Valsartan treatment group could reduce the expression of CTGF mRNA in rat myocardium (*P* < 0.01), but had no significant effect on TGF-*β*1 mRNA (*P* > 0.05), while QSYQ group could obviously inhibit the expression of TGF-*β*1 and CTGF mRNA in rat myocardium (*P* < 0.01). At 8 weeks, there was no statistical difference in the expression levels of TGF-*β*1 and CTGF mRNA in each group (*P* > 0.05) (Figure 5).

### 3.6. Effect of QSYQ on Protein Expression of TGF-*β*1 and CTGF

**Figure 6:**
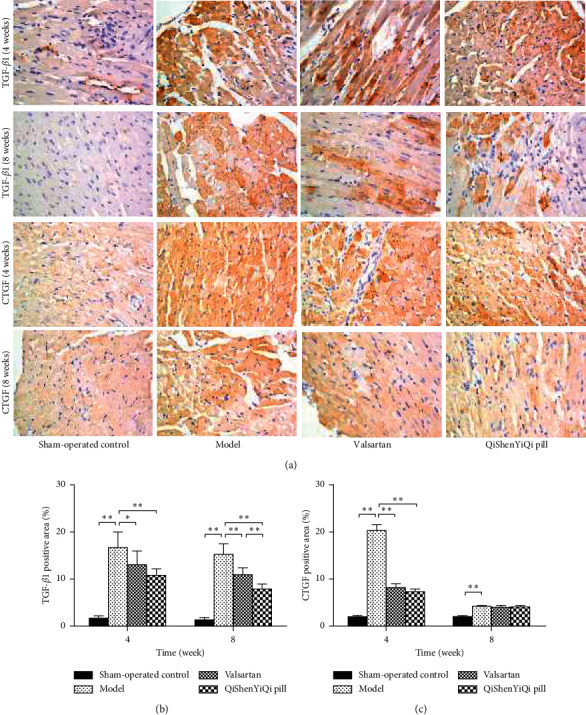
Effect of QSYQ on the protein expression of TGF-*β*1 and CTGF. (a) Representative photomicrograph of immunohistochemical staining of myocardium. (b) The TGF-*β*1 positive area for each group. (c) The CTGF positive area for each group. The percentage of immunohistochemical staining area was quantitatively analyzed by using Image-Pro Plus 6.0 (magnification, × 400). Data are expressed as mean ± SD. ^*∗*^*P* < 0.05 and ^*∗∗*^*P* < 0.01.

Immunohistochemical staining of TGF-*β*1 and CTGF in myocardial tissue showed that a small amount of color change was weak in the sham-operated control group, but in the model group, the brown area was enlarged, the staining was enhanced, and the expression of TGF-*β*1 and CTGF was increased. After the treatment (valsartan and QYSQ), the expression of TGF-*β*1 and CTGF was reduced compared with the model group. Semiquantitative analysis showed that the percentage of positive area of TGF-*β*1 and CTGF proteins in the pressure overload model group was significantly higher than that in the sham-operated group at 4 weeks (*P* < 0.01). Valsartan treatment could reduce the percentage of positive area of TGF-*β*1 and CTGF protein (*P* < 0.05 or *P* < 0.01), while QSYQ group had similar results. At the 8th week, the percentage of positive area of TGF-*β*1 and CTGF protein in the model group was still higher (*P* < 0.01). The positive area of CTGF protein in each group (*P* > 0.05) had no significant difference, but valsartan group could reduce the positive area of TGF-*β*1 protein. QSYQ group showed the same effect as valsartan group, but the decreasing effect was better than valsartan group (*P* < 0.01) (Figure 6).

## 4. Discussion

Hypertension is the most common chronic noncommunicable disease with high morbidity, disability, and mortality. Although the awareness rate, treatment rate, and control rate of hypertension are on the rise, they are still at a low level [21]. In clinical practice, more than 30% of hypertensive patients may have LVH, and the incidence of LVH is positively correlated with the severity of hypertension [22]. Long-term pressure overload leads to excessive deposition of cardiac collagen fibers, increased collagen concentration, unbalanced collagen ratio, and disordered arrangement, resulting in changes in cardiac function and structure, thereby increasing the incidence of cardiovascular events. A meta-analysis of 2449 patients also showed that the risk of total cardiovascular events in patients with left ventricular hypertrophy reversed/persistently normal hypertension decreased by 46% [23]. Therefore, the prevention/reversal of LVH can significantly reduce the risk of cardiovascular events and death and has become one of the hot topics in the field of CVD [24, 25].

Type I and III collagen are the main collagen in the heart, of which type I collagen accounts for 85% and type III collagen for 11%. The collagen content (collagen volume fraction, CVF) in normal myocardial tissue of rats is about 3%–5%. When CVF increases to 8%–12%, the diastolic function of the myocardium is damaged and the systolic function is maintained. However, when the CVF rises to more than 20%, the systolic function of rat myocardium becomes impaired [26]. ANP and *β*-MHC are markers of cardiomyocyte hypertrophy. In pressure-overloaded rats, with the prolongation of intervention time, QSYQ could further reduce myocardial collagen content and CVF, reduce the expression of ANP and *β*-MHC mRNA, downregulate the expression of type I and III collagen, and reduce the degree of myocardial hypertrophy. It was suggested that QSYQ could effectively improve the myocardial hypertrophy of rats with pressure overload and had an effect of resisting myocardial hypertrophy.

Long-term high-load heart work will lead to myocardial hypertrophy, interstitial fibrosis, and microvascular changes and ultimately damage the cardiac contractile function and lead to HF [27]. TGF-*β*1 is closely related to extracellular matrix deposition, and it is recognized as a target for the treatment of organ fibrosis [28]. TGF-*β*1 has multiple physiological functions, which can inhibit inflammation and cell proliferation during normal expression, while overexpression can lead to adverse consequences, such as fibrosis [29]. Pressure overload, myocardial infarction, immune injury, and other stimuli can activate TGF-*β*1 signal and initiate fibrosis, resulting in a large amount of collagen deposition [30]. Clinical studies have shown that the increase of plasma TF-*β*1 levels in hypertensive patients is closely related to hypertension and its target organ damage [31]. Compared with normal blood pressure rats, the expression of TGF-*β*1 in the blood vessels of spontaneously hypertensive rats increased, and the increased expression of TGF-*β*1 could induce the proliferation of vascular smooth muscle cells [32]. Under pressure overload, hypoxia inducible factor 1 (HIF-1) protects the heart and aorta of mice by downregulating the expression of TGF-1-Smad2/3 and TGF-*β*1-ERKl/2 [33]. In addition, fluvastatin can inhibit TGF-*β*1 and induce Smad7 expression in a dose-dependent manner, thereby delaying myocardial hypertrophy and myocardial fibrosis in spontaneously hypertensive rats [34]. The results showed that QSYQ could significantly inhibit the expression of TGF-*β*1 mRNA and reduce the positive area of TGF-*β*1 protein in rat myocardium after intervention for 4 weeks, while reducing the positive area of TGF-*β*1 protein in rat myocardium for 8 weeks. It suggested that the effect of QSYQ on the antihypertrophy of myocardium was related to the expression of TGF-*β*1.

Connective tissue growth factor (CTGF) is a downstream effect factor of TGF-*β*1, which only mediates the negative effect of TGF-*β*1. Therefore, inhibiting CTGF expression may be a new target for the treatment of myocardial hypertrophy. CTGF, as a fibrotic factor, is closely related to the development of multiple organ fibrosis. It is confirmed that TGF-*β* can induce the upregulation of CTGF expression in cardiac myocytes and cardiac fibroblasts, and the upregulation of CTGF is associated with increased expression of fibronectin, type I collagen, and type III collagen [35]. The expression of TGF-*β*1 and CTGF in myocardial infarction model rats showed that TGF-*β*1 was mainly expressed in the onset of myocardial infarction, acute inflammatory response, and postrepair stage of myocardial infraction, while CTGF played a role in the development stage of myocardial fibrosis [36]. TGF-*β*1 can induce the expression of CTGF, and CTGF can enhance the TGF-*β*1 signaling pathway. They interact with each other and enter a vicious cycle, which leads to the accumulation of extracellular matrix and eventually develops into cardiac hypertrophy. In addition, myocardial fibrosis in mdx mice began with the increase of CTGF expression, and the upregulation of CTGF was consistent with the increase of TIMP-1 expression [37]. CTGF promotes the development of myocardial fibrosis by promoting collagen synthesis and inhibiting its degradation. In our study, we found that QSYQ can reduce the expression of CTGF mRNA and protein at 4 weeks. This may be the target of QSYQ intervention in myocardial hypertrophy, and further research is needed.

## 5. Conclusion

QSYQ can reduce the content and volume fraction of myocardial collagen, decrease the mRNA expression of ANP and *β* -MHC, and downregulate the protein expression of collagen type I and III. The results indicate that QSYQ can effectively improve myocardial hypertrophy in pressure-overloaded rats, and its mechanism may be related to the regulation of TGF-*β*1 and CTGF.

## Data Availability

The data used to support the findings of this study are included within the article.
